# Metagenomic analysis of tongue samples from healthy subjects identifies distinct microbiome orotypes

**DOI:** 10.1080/20002297.2026.2687934

**Published:** 2026-07-11

**Authors:** Sadeq Ali Al-Maweri, Raidan Ba-Hattab, Abeer Alomairi, Abdullah Syed, Khalid Azouni, Nadeen Batta, Fathiya Almeer, Rami Assad, Alghalia Al-Mansoori, Nahla O. Eltai, Najah Al-Hashimi, Nezar Noor Al-Hebshi, Abeer A. Almashraqi

**Affiliations:** a College of Dental Medicine, QU Health, Qatar University, Doha, Qatar; b Primary Health Care Corporation, Doha, Qatar; c Hamad Medical Corporation, Doha, Qatar; d Biomedical Research Center, Qu Health Sector, Qatar University, Doha, Qatar; e Oral Microbiome Research Laboratory, Department of Oral Health Sciences, Maurice H. Kornberg School of Dentistry, Temple University, Philadelphia, PA, USA

**Keywords:** Oral microbiome, shotgun sequencing, phageome, bacteriome, orotypes

## Abstract

**Background:**

The tongue dorsum harbors a complex microbiome that remains incompletely characterized.

**Objective:**

This study aimed to characterize the tongue microbiome—including its phageome—in a healthy Qatari population.

**Design:**

Shotgun metagenomic sequencing was performed on tongue-coating samples from 92 systemically healthy adults to comprehensively profile the bacteriome, phageome and functional potential of the tongue microbiome.

**Results:**

Taxonomic profiling revealed a predominantly bacterial community (>99%) dominated by *Veillonella, Streptococcus, Neisseria, Rothia, Prevotella, Haemophilus* and *Pauljensenia*. Among low-abundance domains, the fungus *Saccharomyces* and the protist *Entamoeba* were most prevalent. Dirichlet–multinomial mixture clustering identified three distinct bacterial ‘orotypes’ (C1–C3) showing significant compositional separation (PERMANOVA, *p* = 0.001) and alpha diversity differences at both genus and species levels. A major compositional gradient involved enrichment of *Neisseria* and *Haemophilus* in C2, their absence in C3 and intermediate representation in C1. Functional profiling revealed a conserved core of housekeeping pathways across orotypes, wherease adaptive functionsdiffered across orotypes, particularly in the Neisseria/Haemophilus-enriched C2 orotype. The phageome was dominated by Uroviricota (class Caudoviricetes).

**Conclusion:**

The findings identify distinct tongue microbiome orotypes with conserved core functions, divergent taxonomic and metabolic profiles, and provide new insights into the tongue phageome, establishing a foundation for investigating their roles in health.

## Introduction

The oral cavity harbors one of the most diverse microbial ecosystems in the human body, second only to the gut. It comprises bacteria, archaea, viruses, fungi and protozoa, with more than 700 bacterial species identified to date [1,2]. Approximately 96% of oral bacteria belong to six major phyla: *Actinobacteria*, *Bacteroidetes*, *Fusobacteria*, *Firmicutes*, *Spirochetes* and *Proteobacteria* [3]. The oral cavity contains multiple anatomically distinct niches—including the hard and soft palate, supragingival and subgingival plaque, tongue and tooth surfaces—each providing a unique environmental that shape microbial colonization and community structure [1].

Among these niches, the tongue represents a particularly distinctive microbial habitat. The dorsal tongue surface is heavily papillated, which creates a protected microenvironment for microbial colonization [4]. The tongue dorsum is among the most densely colonized mucosal surfaces of the oral cavity and harbors a diverse and structured microbial community serving as a reservoir for salivary microbiota [5,6]. As a highly vascularized muscular organ containing lymphoid tissues such as lingual tonsils, the tongue forms an important interface between resident microbiota and the host immune system. Alterations in the tongue microbiome have been associated with oral conditions such as halitosis, geographic tongue and taste disorders [7–9]. Emerging evidence associates alterations in tongue microbial communities with aspiration pneumonia and certain malignancies as well as blood pressure regulation mediated by nitrate-reducing bacteria [10–12].

Population-level studies such as the human microbiome project consortium provided the first comprehensive 16S rRNA gene–based characterization of the healthy tongue dorsum microbiome compared to other sites [5,13]. Subsequent investigations further described spatial organization of the tongue microbiome using multiplexed fluorescence spectral imaging, also based on 16S rRNA gene sequences [14]. More recently, large-scale oral metagenomic studies have reconstructed extensive metagenome-assembled genome (MAG) catalogs from thousands of oral samples, including tongue specimens [15]. Shotgun metagenomic approaches enable species- and strain-level resolution, functional profiling and multi-kingdom characterization overcoming key limitations of 16S-based surveys [16]. Despite these advances, comprehensive metagenomic analyzes specifically centered on the healthy tongue are nearly lacking. Notably, no study to date has characterized the tongue microbiome of healthy Arab populations using metagenomics. Considering the potential impact of genetic background, cultural practices, dietary habits and environmental factors on microbial community structure, population-specific studies are essential [17].

The oral phageome, in particular, has emerged as an important yet underexplored component of the oral ecosystem [18]. Saliva contains up to 10⁵ virus-like particles per microliter, the majority of which are bacteriophages [19,20]. Phages influence bacterial population composition and stability and play a role in horizontal gene transfer, yet their role within the healthy tongue microbiome remains poorly defined. Considering the gaps highlighted above, the present study aimed to characterize the healthy tongue microbiome—including its phageome—in a Qatari population using whole metagenomic sequencing.

## Materials and methods

### Ethical approval

The study received ethical approval from the Institutional Review Board of Qatar University (IRB-QU; Ref. No. QU-IRB 344/2024-EM), the Primary Health Care Corporation (PHCC; Ref. No. BUHOOTH-D-23-000109) and the Qatar University Institutional Biosafety Committee (IBC-QU; Ref. No. QU-IBC-039/2024). The study was conducted in compliance with the ethical principles of the World Medical Association Declaration of Helsinki (2013). Written informed consent was obtained from all participants.

#### Study design, setting and participant recruitment

This cross-sectional study included ninety-two systemically healthy subjects recruited as part of the Q-TONGUE cohort (Tongue-coating microbiome in health and hypertension in Qatar). The recruitment of the study participants was carried out at Primary Health Care Cooperation (PHCC) in Qatar from July 2024 to May 2025. PHCC has multiple centers covering the entire country. These centers are divided into three main regions: Western, Northern and Central. Participants were recruited from the large centers in each region to ensure that the samples reflected the Qatari population.

To initiate recruitment, the IT department at PHCC extracted data of systemically healthy individuals from electronic data records (EDR) identified based on the study's inclusion and exclusion criteria. Then, a Family Medicine Specialist (AS) with more than 20 years of experience reviewed the data and identified eligible participants.

#### Inclusion and exclusion criteria

Participants were eligible for inclusion if they met the following criteria: adults aged ≥ 18 years; Qatari nationality; and systemically healthy. Participants were excluded if they were pregnant or lactating; had current or recent (<3 months) use of antibiotics or antifungals; had recent use of mouthwashes; were receiving immunosuppressive medications; had acute infections; had a diagnosis of chronic systemic diseases (including diabetes, autoimmune disorders, cancer, or renal disease); or had received periodontal disease treatment within the preceding six months.

#### Metadata collection and oral health evaluation

Participant metadata were collected using a standardized, structured questionnaire administered by a trained dentist. Information included demographic characteristics, socioeconomic status, lifestyle factors (such as smoking status and mouthwash use) and relevant medical history, including medication use. Medical history and medication use were cross-checked with the EDR and, in cases of conflicts, reconciled by the family medicine specialist. Calibrated dental practitioners (AA, KA, NB) conducted a comprehensive dental examination, including assessment of dental caries, periodontal health status, tooth loss and soft tissue status. Periodontal health status was assessed according to the 2017 World Workshop on the Classification of Periodontal and Peri-Implant Diseases and Conditions [21].

#### Nitrate intake assessment

Dietary nitrate intake was estimated using a weighted scoring system based on a food frequency questionnaire recording ‘Yes/No’ consumption of selected foods. Foods were assigned scores according to their relative nitrate contribution, with leafy green and root vegetables receiving a score of 3, moderate sources a score of 2, and minor sources a score of 1. A total nitrate intake score (NIS) was calculated for each participant by summing the scores of consumed items. Participants were classified into high- and low-nitrate diet groups using the median NIS as the cutoff [22–25]. Given that scoring was based on food-frequency responses rather than quantitative dietary measurements, NIS should be interpreted as a surrogate estimate, not an absolute measurement of nitrate intake.

#### Tongue dorsum sampling

Microbial samples were collected from the dorsal surface of the tongue using flocked swabs (SK2; Isohelix, UK). Sampling was performed using firm, overlapping strokes applied 10 times in one direction, followed by a 180° rotation of the swab and an additional 10 strokes in the opposite direction. Swabs were placed in sterile, barcoded 2-mL tubes prefilled with Tris–EDTA buffer (pH 8.0; Fisher Scientific, USA), transported to the laboratory on dry ice and stored at 4 °C for a maximum of 10 days prior to DNA extraction (this was done to avoid freeze–thaw–induced microbial DNA release, which could lead to unintended loss during the host DNA depletion step).

#### DNA extraction and sequencing

Each swab was swirled in the TE buffer to release the sample, transferred into a spin basket (Promega, USA) and centrifuged to recover absorbed buffer and any remaining sample material; the recovered eluate was then pooled with the original suspension. DNA was subsequently extracted using the QIAamp Microbiome DNA Kit (Qiagen, USA) according to the manufacturer's instructions, which include a host DNA depletion step followed by mechanical and chemical lysis of microbial cells. DNA yield and purity were assessed using a NanoDrop spectrophotometer (Thermo Fisher Scientific, USA).

Shotgun metagenomic sequencing was performed at CosmosID (MD, USA). Briefly, DNA libraries were prepared using the Watchmaker DNA Library Prep Kit (Watchmaker Genomics, USA). Genomic DNA was enzymatically fragmented using the Frag/AT Buffer and Enzyme Mix, followed by adapter ligation with IDT xGen UDI primers and IDT Stubby Adapters (Integrated DNA Technologies, USA). Libraries were amplified by 7 cycles of PCR and purified using CleanNGS magnetic beads (CleanNA, Netherlands). Libraries were sequenced on the Novaseq X sequencer (Illumina, USA) with 2 × 150 bp paired-end chemistry, targeting approximately 20 million read pairs per sample.

#### Multi-kingdom taxonomic and functional profiling

Unassembled whole-genome shotgun sequencing reads were analyzed using the Kepler™ multi-kingdom microbiome profiling pipeline available through the Cosmos-Hub platform (https://cosmos-hub.com/). Kepler is a host-independent, reference-based algorithm for detection and quantification of microbial taxa, including bacteria, archaea, fungi, protists and non-phage viruses. Taxonomic profiling relies on a precomputed reference resource (GenBook™), which contains more than 150,000 curated genomes and gene sequences representing over 30,000 microbial species. Database construction involves splitting genomes into variable-length k-mer biomarkers that capture shared and taxon-specific genomic signatures, structured into a phylogenetic-like tree. Sample-level classification is conducted in two steps. First, sequencing reads are fragmented into k-mers and screened against the GenBook™ biomarker database through exact matching to rapidly identify a subset of candidate reference taxa. Second, a probabilistic, edit-distance–based Smith–Waterman alignment algorithm is used for abundance estimation and taxonomic refinement to generate kingdom-to-strain profiles [26].

Microbial functional potential was inferred from shotgun metagenomic data using HUMAnN 3 [27]. Briefly, sequencing reads were first aligned at the nucleotide level to the ChocoPhlAn pangenome database using Bowtie2 [28] to identify gene families. Reads that did not map at the nucleotide level were subsequently translated and aligned against the UniRef90 protein reference database [29] using DIAMOND [30] to capture additional functional features. Gene family abundance profiles were then aggregated into higher-level functional annotations, including Enzyme Commission [31], PFam protein families [32], MetCyc metabolic pathways [33] and Gene Ontology biological processes [34].

#### Characterization of bacteriophage communities

Bacteriophage communities (phageome) were profiled using the Clinical Human-Associated Microbiome Profiling (CHAMP) framework, which is based on a curated, non-redundant collection of high-confidence, annotated human-associated viral genomes representing 64,003 viral operational taxonomic units (vOTUs), the majority of which represent bacteriophages [35]. Shotgun sequencing reads were mapped to the vOTU reference database using a K-mer alignment (KMA) algorithm [36], with additional filtering to prevent cross-mapping to bacterial genomes. Viral taxa were considered detected based on a minimum of five paired-end reads and stringent sequence identity and genome coverage thresholds (≥99% nucleotide identity with ≥ 5% genome coverage, or ≥ 95% identity with ≥ 30% genome coverage). Phage abundance was estimated from uniquely mapped read pairs normalized by genome length. Putative bacterial hosts for each vOTU were assigned using precomputed annotations within the CHAMP framework, derived from complementary CRISPR spacer matching and k-mer similarity analyses against extensive collections of reference and metagenome-assembled bacterial genomes. Host predictions, therefore, reflect reference-supported associations independent of the bacterial taxonomic profiles observed in the study samples by Kepler.

#### Microbiome data summarization

All data analyses described in this and subsequent sections were performed in R 4.5.1 using the Phyloseq [37], microbiome [38] and related visualization packages. For community-level visualization, bacterial relative abundance profiles were summarized at multiple taxonomic ranks (phylum, genus, species and strain) to depict average bacteriome composition across samples. Low-abundance domains were summarized by prevalence and relative abundance. Inter-individual variation in dominant bacterial taxa was assessed by hierarchical clustering of samples using Euclidean distance and average linkage, based on the 15 most abundant genera and, separately, the 25 most abundant species and visualized as heatmaps. Phageome composition and predicted bacterial host profiles were similarly summarized at relevant taxonomic ranks using relative abundance estimates.

#### Microbiome analysis by metadata

Differences in alpha diversity, measured as species richness and Shannon Index, across categorical variables, including gender, smoking status, mouthwash use, periodontitis status and nitrate intake category, were evaluated using Wilcoxon rank-sum test**s**. Associations between alpha diversity and continuous variables, including age and NIS, were assessed using Spearman's rank correlation. Beta diversity was assessed using principal component analysis (PCA) based on Aitchison distances computed from centered log-ratio (CLR)–transformed data. Differences in overall community composition associated with each variable were evaluated using permutational multivariate analysis of variance (PERMANOVA). Associations between microbial species and metadata variables were assessed with the MaAsLin3 [39] package in R, using CLR-transformed data and including all variables as covariates.

#### Dirichlet–multinomial mixture modeling of microbiome profiles

To identify potential microbiome community types (orotypes) in tongue-coating microbiome profiles—analogous to enterotypes described in the gut microbiome—Dirichlet–multinomial mixture (DMM) modeling [40] was applied to genus-level and species-level count tables using the DirichletMultinomial R package [41]. For each taxonomic rank, models with k = 1–5 components were fitted. Model selection was performed using the Laplace approximation, and the optimal number of components was chosen based on the minimum Laplace value (which resulted in k = 3 for both genus- and species-level analyzes). Posterior mixture probabilities were computed, and each sample was assigned to the DMM cluster (hereafter referred to as ‘orotype’) for which it had the highest posterior probability. To identify taxa driving each orotype, the fitted Dirichlet concentration parameters (*α*) were extracted and normalized within each orotype (α/Σα), yielding Dirichlet weights that represent the model-implied expected contribution of each taxon to that orotype. Differences in beta diversity among orotypes were assessed by PCA and PERMANOVA as described above. Permutational analysis of multivariate dispersions (PERMDISP) was performed to assess within-orotype heterogeneity. Differences in alpha diversity were evaluated using Kruskal–Wallis tests, followed by pairwise Wilcoxon rank-sum tests with Benjamini–Hochberg (BH) correction for multiple comparisons.

For each functional category, differential abundance of individual features across orotypes was assessed using the Kruskal–Wallis test. Prior to statistical testing, features detected in fewer than 10% of samples or exhibiting no variability across samples were excluded. Resulting *p*-values were adjusted for multiple comparisons using the BH false discovery rate (FDR) procedure, and features with FDR ≤ 0.05 were considered statistically significant.

## Results

### Overall multi-kingdom microbial profiles

This study comprised 92 participants (41 (44.57%) females and 51 (55.43%) males) with a mean age of 41.23 ± 7.89. The participants' clinical characteristics are summarized in Table 1.

**Table 1.** Characteristics of the study participants.

Variables	Participants (*n* = 92)
Age (years)	41.23 ± 7.89
Gender	
Female	41 (44.6%)
Male	51 (55.4%)
Plaque index (average)	0.14 ± 0.12
Periodontitis	
No	60 (65.2%)
Yes	32 (34.8%)
Smoking	
No	68 (73.9%)
Yes	23(25.0%)
Nitrate intake score	
Low-nitrate diet	36 (39.1%)
High-nitrate diet	56 (60.9%)
Mouthwash use	
No	85 (92.4%)
Yes	7 (7.6%)

**Figure 1.**
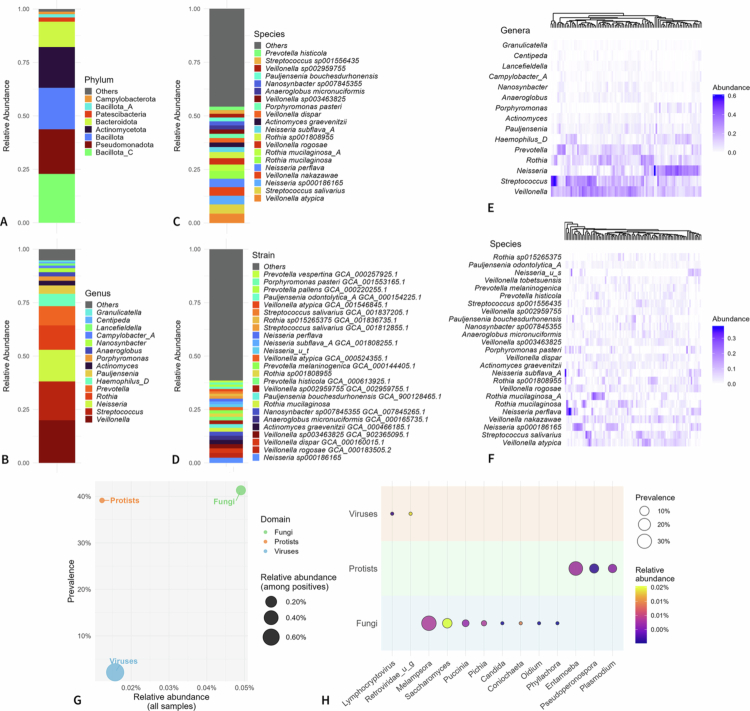
Multi-kingdom tongue coating microbiome composition in healthy individuals. DNA was extracted from tongue swab samples collected from healthy participants and subjected to shotgun metagenomic sequencing on the NovaSeq X platform (2 × 150 bp). Sequencing reads were taxonomically classified to the strain level using the Kepler microbiome profiler against a comprehensive reference database encompassing bacterial, eukaryotic and non-phage viral genomes. The most abundant bacterial phyla (A), genera (B), species (C) and strains (D) are shown as stacked column plots. Inter-individual variation in community structure is visualized using heatmaps of the top 15 genera (E) and top 25 species (F). Samples were hierarchically clustered using Euclidean distance and average linkage, and taxa are ordered by descending total relative abundance. (G) Bubble plot summarizing the prevalence and abundance of low-abundance microbial domains (fungi, protists and non-phage viruses) across samples, where bubble size reflects mean abundance among positive samples. (H) Prevalence and relative abundance of key genera within each low-abundance domain.

The tongue microbiome was dominated by bacteria, accounting for 99.92% of the microbial community. The average bacteriome composition at different taxonomic levels is presented in Figure 1A–D. At the phylum level, the most abundant phyla were Bacillota (including clades A and C), *Pseudomonadota*, *Actinomycetota* and *Bacteroidota*. At the genus level, more than 75% of the bacterial community was made up of *Veillonella, Streptococcus, Neisseria, Rothia, Prevotella* and *Hemophilus*. At the species level, 20 species accounted for more than 50% of the average bacteriome, with *Rothia mucilaginosa (including clade A) being the most dominant, followed by Veillonella atypica*, *Streptococcus salivarius*, *Neisseria* sp. 000186156, *Veillonella nakazawae* and *Neisseria perflava*. Greater diversification was observed at the strain level, with 25 strains collectively accounting for only around 35% of the bacteriome on average.

Hierarchical clustering of samples based on the relative abundance of the top 15 genera (Figure 1E) and top 25 species (Figure 1F) revealed that although dominant taxa were shared across individuals, their relative rankings varied substantially among subjects, resulting in apparent grouping into 2–3 clusters. To formally evaluate this community structuring, Dirichlet multinomial mixture (DMM) clustering was subsequently performed (see below).

Low-abundance microbial domains, including fungi, protists and non-phage viruses, represented 0.08% of the average microbiome, with fungi being the most abundant among them (Figure 1G). Fungi and protists exhibited relatively high prevalence (~40%), whereas non-phage viruses were detected in only two samples but were relatively abundant within those samples (~0.60%). At the genus level, *Saccharomyces* and *Entamoeba* were the most abundant fungal and protist taxa, respectively (Figure 1H).

### Clinical factors modestly influence the tongue microbiome

**Figure 2.**
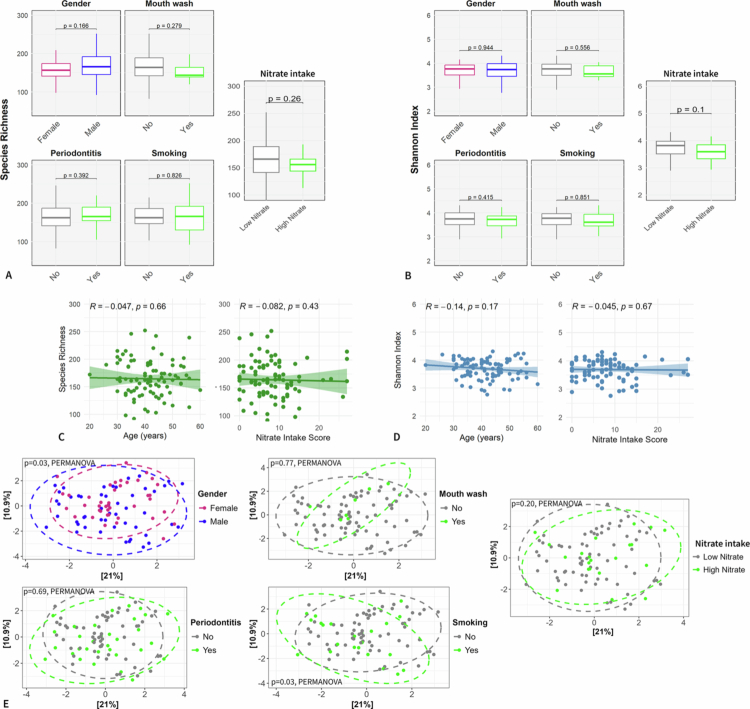
Tongue microbiome diversity in relation to clinical factors. Alpha diversity (species richness and Shannon index) and beta diversity were evaluated in relation to key metadata variables, including gender, age, mouthwash use, periodontal health status, smoking status and nitrate intake. Differences in alpha diversity across categorical variables were assessed using Wilcoxon rank-sum tests (A–B), while associations with continuous variables were evaluated using Spearman rank correlation (C–D). Beta diversity was visualized using principal component analysis (PCA) based on Aitchison distances computed from centered log-ratio (CLR)–transformed data (E). Differences in overall community composition were assessed using permutational multivariate analysis of variance (PERMANOVA).

Species richness and Shannon index showed no significant differences across categorical clinical variables, including gender, smoking, mouthwash use and periodontal status (Figure 2A–B). Similarly, age and nitrate intake score (NIS) exhibited weak and non-significant correlations with alpha diversity (Figure 2C–D), indicating relative stability of within-sample microbial diversity.

Beta diversity analysis based on Aitchison distances demonstrated that, in univariate PERMANOVA models, overall microbial composition was significantly associated with smoking status and gender, but not with age, mouthwash use, periodontal status, or NIS (Figure 2E). However, these associations were no longer significant after adjusting for analysis, suggesting that their effects on overall community structure were modest.

**Figure 3.**
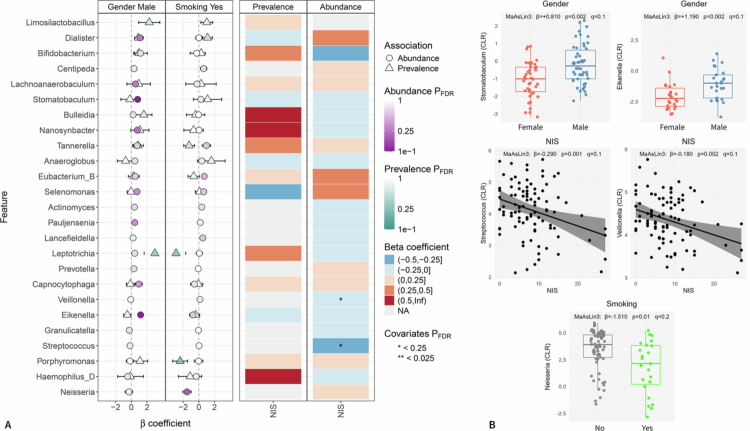
Modeling of taxa associated with clinical factors. Centered log-ratio (CLR)–transformed data were analyzed using the MaAsLin3 package in R to identify microbial genera associated with key metadata variables, including gender, smoking status and nitrate intake score (NIS). Statistical significance was defined at a false discovery rate (FDR) ≤ 0.25. (A) Associations for 25 genera are shown. For the two variables exhibiting the strongest associations, results are visualized as coefficient plots, while associations with the remaining variables are displayed as heatmaps. (B) Boxplots and correlation plots for selected genera showing significant associations with clinical factors; corresponding MaAsLin3 statistics are indicated.

Taxon-level multivariate modeling (MaAsLin3) identified targeted associations with specific genera (Figure 3), with smoking and gender showing the strongest effects. Smoking was associated with depletion of *Neisseria* and lower prevalence of *Leptotrichia* and *Porphyromonas*. Males exhibited a higher abundance of *Stomatobaculum* and *Eikenella*, along with a greater prevalence of *Leptotrichia*. Higher NIS was associated with lower relative abundance of *Streptococcus* and *Veillonella*.

### Dirichlet multinomial mixture (DMM) analysis identifies distinct tongue microbiome orotypes

Since hierarchical clustering based on overall taxonomic profiles suggested ecological groupings within the cohort (Figure 1E and F), we applied Dirichlet–multinomial mixture (DMM) modeling to identify potential tongue microbiome orotypes.

**Figure 4.**
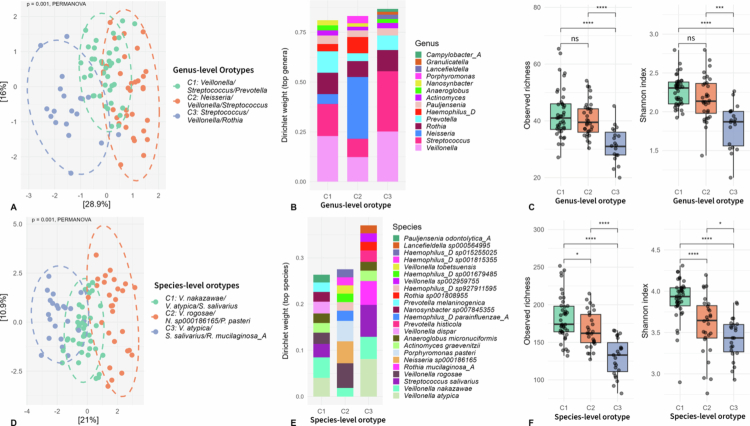
Microbiome orotypes identified in healthy tongue-coating samples. Dirichlet–multinomial mixture (DMM) models were fitted to genus-level and species-level count data, and the optimal cluster number (k = 3) was selected using the minimum Laplace approximation. Each sample was assigned to the DMM cluster (orotype) with the highest posterior probability. (A) Principal component analysis (PCA) by genus-level orotype (C1–C3) based on Aitchison distances computed from centered log-ratio (CLR)–transformed counts. Separation was evaluated by PERMANOVA (B) Top genera driving each genus-level orotype, ranked by Dirichlet weights (within-orotype normalized Dirichlet parameters, α/Σα), representing the predicted contribution of each genus to the orotype. (C) Alpha diversity across genus-level orotypes, shown as observed richness and Shannon index; groups were compared using Kruskal–Wallis test, followed by pairwise Wilcoxon rank-sum tests with Benjamini–Hochberg correction for multiple comparisons; significant pairwise comparisons are indicated. (D-F) Corresponding PCA plot, Dirichlet-weight profiles and alpha diversity comparisons for species-level orotypes.

At the genus level, DMM analysis resolved three orotypes (C1–C3) that were clearly separated in beta-diversity analysis by PERMANOVA, both overall and in pairwise comparisons (all *p* < 0.001). (Figure 4A), indicating significantly distinct community compositions. Dirichlet weights identified the dominant genera characterizing each orotype (Figure 4B). C1 was dominated by *Veillonella*, followed by *Streptococcus*, *Prevotella* and *Rothia*; C2 was primarily driven by *Neisseria* and *Haemophilus*, with additional contributions from *Veillonella*, *Streptococcus* and *Rothia*; and C3 showed substantial overrepresentation of *Streptococcus*, followed by *Veillonella*, *Rothia* and *Prevotella,* with a lack of contribution from *Neisseria or Haemophilus*. Alpha diversity differed significantly among orotypes (*p* < 0.0001) (Figure 4C), with C1 exhibiting the highest species richness and Shannon diversity, whereas C3 consistently showed the lowest diversity.

Species-level DMM analysis similarly identified three distinct orotypes (C1–C3) demonstrating significant compositional separation, both overall and in pairwise comparisons (all *p* < 0.001) (Figure 4D). They showed moderate concordance with the genus level (adjusted rand index [ARI] = 0.51). Species-level taxa driving each orotype based on Dirichlet weights are presented in Figure 4E. C1 was driven by *V. nakazawae*, *V. atypica*, *S. salivarius, other Veillonella* spp. and *Prevotella melaninogenica*; C2 showed high contributions from *Veillonella rogosae* and *Neisseria* sp. 000186165, *Porphyromonas pasteri and Haemophillus* spp..; and C3 was dominated by *V. atypica*, *S. salivarius* and *R. mucilaginosa,* followed by *V. nakazawae* and *Prevotella histicola*, with no contribution from *Neisseria* or *Haemophilus* spp. Alpha diversity patterns at the species level mirrored those observed at the genus level (Figure 4F), with C1 displaying the highest diversity and C3 the lowest, reinforcing the robustness and consistency of the DMM-derived ecological groupings.

Assessment of multivariate dispersion (PERMDISP) showed no significant differences between orotypes at the genus level, whereas species-level profiles exhibited significant dispersion differences, primarily involving the C2 orotype, indicating greater within-orotype heterogeneity at finer taxonomic resolution.

**Figure 5.**
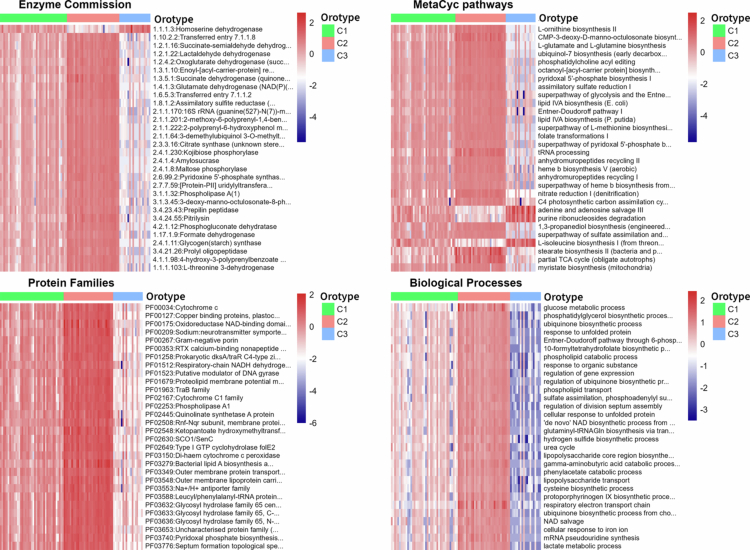
Functional signatures distinguishing genus-level microbiome orotypes. Functional profiling of metagenomic data was performed using HUMAnN 3.0. Heatmaps showing the top differentially abundant functional features across genus-level orotypes C1–C3. Panels show (A) enzyme commission numbers, (B) MetaCyc metabolic pathways, (C) Pfam protein families and (D) gene ontology biological processes. For each functional category, features were tested for differential abundance across orotypes using Kruskal–Wallis rank-sum tests, with *p*-values adjusted using the Benjamini–Hochberg false discovery rate (FDR) procedure. The 30 features with the lowest FDR values in each category are shown. Values represent log₁₀-transformed relative abundances (log₁₀[abundance + 10⁻⁶]) standardized per feature (row-wise z-score). Feature names were truncated to 40 characters for readability.

### Tongue microbiome oryotypes are largely independent of clinical factors

Associations between the orotypes and demographic or clinical variables are summarized in Table 2 and Supplementary Table 1. At both genus and species levels, no demographic or clinical variable differed significantly across orotypes, indicating that the identified orotypes were largely independent of measured metadata. At the genus level, however, smoking status showed a borderline association (*p* = 0.093), with C2 containing proportionally fewer smokers compared to C1 and C3.

**Table 2.** Association of clinical factors with genus-level DMM clusters (orotypes).

Variable	C1 (*n* = 41)	C2 (*n* = 32)	C3 (*n* = 19)	*p*-value
Gender				
Female	16 (39.0%)	17 (53.1%)	8 (42.1%)	0.471
Male	25 (61.0%)	15 (46.9%)	11 (57.9%)	
Mouthwash				
No	37 (90.2%)	30 (93.8%)	18 (94.7%)	0.885
Yes	4 (9.8%)	2 (6.2%)	1 (5.3%)	
Nitrate intake				
High	6 (14.6%)	11 (34.4%)	4 (21.1%)	0.139
Low	35 (85.4%)	21 (65.6%)	15 (78.9%)	
Periodontitis				
No	27 (65.9%)	21 (65.6%)	12 (63.2%)	0.978
Yes	14 (34.1%)	11 (34.4%)	7 (36.8%)	
Smoking				
No	28 (70.0%)	28 (87.5%)	12 (63.2%)	0.0932
Yes	12 (30.0%)	4 (12.5%)	7 (36.8%)	
Age Median (IQR)	41 (35–46)	42 (36–46)	40 (36–46)	0.738
NIS Median (IQR)	7 (5–10)	8 (5–12)	7 (4–10)	0.637
Plaque index Median (IQR)	0.1 (0.1–0.2)	0.1 (0.0–0.2)	0.2 (0.0–0.2)	0.148

• Categorical variables shown as counts (% within cluster). • Continuous variables shown as median (IQR). • *p*-values: Chi-square, Fisher's exact, or Kruskal–Wallis as appropriate.

### Tongue microbiome oryotypes share core functions but exhibit distinct functional enrichment

Next, we assessed differences in functional potential among the genus-level orotypes. Global heatmap visualization of the most abundant functional features did not show sharp categorical segregation; instead, it revealed a largely conserved functional backbone across orotypes (Supplementary Figure 1). Highly abundant functions were dominated by core housekeeping pathways, including DNA replication and repair (e.g. DNA helicase, DNA-directed polymerase, DNA repair), transcription and translation machinery (e.g. RNA polymerase, ribosomal functions, elongation factors), central carbohydrate metabolism (e.g. glycolysis and glucose metabolic processes) and amino acid biosynthesis pathways (including branched-chain and aromatic amino acids).

In contrast, differential abundance analysis identified distinct functional enrichments across the orotypes, with overall patterns of highest enrichment in *Neisseria*-driven C2, intermediate levels in C1 and broad depletion in C3 across enzyme, pathway, protein-family and biological-process annotations (Figure 5). Differential features distinguishing C2 were primarily related to respiratory/redox functions (e.g. ubiquinone biosynthesis and electron transport), porphyrin/heme metabolism and cell-envelope/outer-membrane functions (including lipid A/LPS-related pathways, porins and transport systems), along with enrichment of cofactor biosynthesis (e.g. pyridoxal phosphate, folate and NAD pathways) and select nitrogen/sulfur metabolism functions (including nitrate reduction and sulfate assimilation). In contrast, C3 showed relative depletion across these modules, consistent with a comparatively reduced functional repertoire.

### Tongue phageome composition and predicted bacterial host profiles

**Figure 6.**
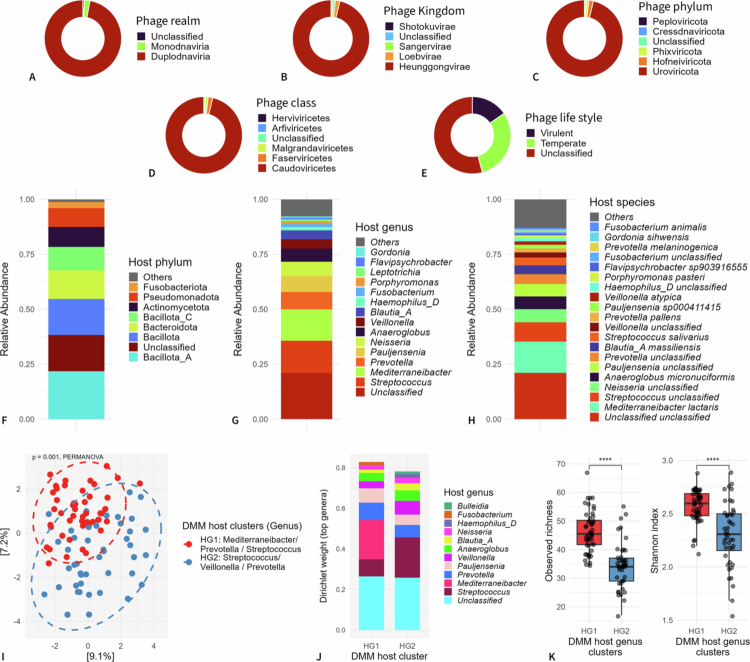
Tongue phageome composition and predicted bacterial host profiles. Shotgun metagenomic reads were mapped to a curated reference database of bacteriophage vOTUs within the CHAMP framework to profile the tonguephageome and its predicted bacterial hosts. The figure provides an exploratory ecological overview of tongue-associated phage communities and predicted host-associated patterns within the healthy tongue microbiome, complementing the bacterial orotype analysis presented in the study. (A–E) Phage relative abundance profiles summarized as stacked bar plots at the realm, kingdom, phylum, class and lifestyle levels, respectively. (F–H) The corresponding predicted bacterial host profiles of detected phages, summarized as stacked bar plots at the phylum, genus and species levels. Dirichlet–multinomial mixture (DMM) modeling applied to host genus–level profiles identified two host-associated DMM clusters (HG1 and HG2) based on the minimum Laplace approximation. (I–K) Cluster-level characterization, including beta diversity separation (I), top host genera driving each cluster ranked by Dirichlet weights (J) and comparisons of alpha diversity (observed richness and Shannon index) between clusters.

Viral sequences almost exclusively matched double-stranded DNA bacteriophages (phageome), whose taxonomic profiles are summarized in Figure 6A–D. At the realm level, the phageome was dominated by Duplodnaviria, with only minor contributions from Monodnaviria. Nearly all classified phages belonged to the kingdom Heunggongvirae, and this pattern continued at deeper taxonomic levels, where Uroviricota represented the dominant phylum while class Caudoviricetes comprised nearly the entire classified community. vOTUs were mostly unclassified at lower taxonomic levels. Viral lifestyles could not be assigned for more than 50% of detected phages; however, among those with predicted lifestyles, temperate phages were more abundant than virulent phages.

Predicted bacterial hosts of detected phages are shown in Figure 6F–H at the phylum, genus and species levels. A substantial proportion (~25%) of host bacteria remained unclassified across taxonomic levels. Among classified hosts, the profiles were dominated by Bacillota (including clades A and C), followed by *Bacteroidota*, *Actinomycetota*, *Pseudomonadota* and *Fusobacteriota* at the phylum level. The most abundant predicted host genera included *Streptococcus, Mediterraneibacter, Prevotella, Pauljensenia, Neisseria* and *Anaeroglobus*, while dominant host species included *Mediterraneibacter lactaris, Anaeroglobus micronuciformis, Streptococcus salivarius* and *Blautia_A massiliensis*, in addition to several unclassified *Streptococcus, Prevotella, Pauljensenia* and *Neisseria* species.

DMM clustering applied to host-associated bacterial genera identified two distinct host clusters (HG1 and HG2) (Figure 6I–K). These clusters showed clear separation in beta-diversity space (PERMANOVA, *p* = 0.001). HG1 was driven with *Mediterraneibacter* with contribution from *Prevotella*, *Streptococcus* and *Pauljensenia*. And exhibited significantly higher richness and Shannon diversity. In contrast, HG2 was dominated by *Streptococcus* followed by *Veillonella*, and *Prevotella,* with no contribution from *Mediterraneibacter*, displaying lower alpha diversity.

## Discussion

To the best of our knowledge, this is the first shotgun metagenomic, strain-level characterization of the healthy tongue microbiome, integrating bacteriome, phageome, functional profiling and community state analyses, and the first such study conducted in an Arab Qatari population. A key and novel finding of this study was the identification of distinct clusters of the tongue microbiota—termed *orotypes*—analogous to the enterotypes described in the gut microbiome [42]. These orotypes exhibited distinct compositional and functional characteristics, suggesting alternative ecological configurations of the tongue microbial community. The study also characterized the tongue phageome, which was composed almost entirely of double-stranded DNA phages and revealed two distinct predicted bacterial host-associated community patterns.

The average tongue microbiome was dominated by *Veillonella, Streptococcus, Neisseria, Rothia, Prevotella, Haemophilus* and *Pauljensenia* (a reclassified group previously included under *Actinomyces*), listed in the order of relative abundance. The same taxa have been reported among the most dominant genera of the tongue microbiome, although in a different relative order, in previous studies [5,14,43,44]. Members of the phylum Fusobacteriota, particularly genera *Leptotrichia* and *Fusobacterium*, were substantially underrepresented in our cohort relative to previous studies, including a study in a comparable population in Saudi Arabia [45]. While this finding may be explained, at least in part, by methodological differences, e.g. metagenomic vs. amplicon sequencing, it may also reflect a population-specific feature of the Qatari cohort, highlighting potential geographic variation in the oral microbiome. The low fungal and protist signal observed may reflect true low abundance in healthy tongue biofilms, although contributions from sequencing depth limitations cannot be excluded.

Interestingly, alpha and beta diversity metrics did not reveal significant differences across demographic or clinical variables, except for modest effects by smoking and gender, suggesting a degree of ecological stability in the tongue microbiome under healthy conditions, consistent with the known resiliency of the oral microbiome [46,47]. Still, differential abundance analysis identified targeted microbiome differences associated with smoking and gender, consistent with the existing literature [44,48–51]. One notable finding was the depletion of *Neisseria* among smokers; this is consistent with findings from large-scale studies [50,51] and may have clinical implications, as *Neisseria* are known to contribute to nitrate reduction, a pathway implicated in the regulation of blood pressure [12,52].

Beyond overall community composition, Dirichlet–multinomial mixture modeling identified, and for the first time, the presence of three distinct bacterial community configurations (orotypes) within the healthy tongue microbiome. Similar community subtypes have been reported for the gut microbiome (enterotypes), representing alternative stable ecological states [42,53]. In the present study, the three tongue microbiome orotypes were primarily defined by differences in the relative abundance of dominant oral genera, particularly *Streptococcus, Veillonella, Neisseria, Rothia,* *Haemophilus* and *Prevotella*. Importantly, these orotypes were reproducible across both genus and species levels, with moderate concordance (ARI = 0.51), indicating robustness of the DMM-derived ecological groupings. Partial discordance likely reflects species-level heterogeneity within dominant oral genera, whereby communities with similar genus-level composition may differ in the relative contributions of individual species. Consequently, species-level clustering may introduce additional community structure not apparent at the genus level. Consistent with this, dispersion analysis indicated greater within-orotype variability at the species level. In contrast, the absence of dispersion differences at the genus level supports that clustering at higher taxonomic resolution reflects true compositional separation rather than differences in variability.

Nevertheless, a key compositional gradient separating the three orotypes at both taxonomic levels was defined by the differential representation of *Neisseria* and *Haemophilus*. These genera were strongly enriched in the C2 orotype, present at intermediate levels in C1, and essentially absent in C3. Similar compositional gradients driven by dominant taxa, for example, *Bacteroides* and *Prevotella* have been described for gut microbiome enterotypes [53]. In the present study, however, the tongue microbiome orotypes showed no association with the clinical factors examined, except formarginal significance with smoking (inverse association with C2), suggesting that these community states may be primarily driven by host genetic factors shaping local microenvironmental conditions on the tongue dorsum, including depth of papillae and coating thickness [6,54–56], which were not evaluated in the current study. These factors can create localized gradients of oxygen availability, nutrient diffusion and host-derived substrates. Together, these findings suggest that the identified orotypes are best interpreted as structured microbiome configurations along a compositional gradient rather than strictly discrete ecological states.

Many of the dominant taxa identified (e.g. *Neisseria*, *Streptococcus*, *Veillonella*) are common across oral niches. Indeed, it has been shown that tongue-coating microbiota closely resemble salivary microbial communities [5]. However, whether the orotypes described here are mirrored in saliva or other oral niches remains to be determined. Likewise, it is unclear whether these orotypes represent tongue-specific ecological states or broader oral microbiome configurations shared across niches. Future studies incorporating paired sampling of tongue coating and saliva or plaque will be important for addressing this question.

The compositional differences across the orotypes were closely mirrored by differences in functional potential. While all orotypes shared a conserved backbone of housekeeping pathways—reflecting core metabolic requirements of the oral microbiota—the *Neisseria/Haemophilus* enriched C2 orotype exhibited the greatest enrichment of pathways related to respiratory and redox metabolism, heme and porphyrin metabolism, cofactor biosynthesis, nitrogen metabolism and cell envelope processes. These functional signatures are consistent with the metabolic capabilities of *Neisseria* and related mucosa-associated bacteria, which are adapted to relatively oxygenated environments and possess extensive redox and transport systems that support respiratory metabolism [57,58]. Notably, enrichment of nitrogen metabolism included pathways associated with nitrate reduction, a function well-documented in oral bacteria such as, *Neisseria* and *Haemophilus* [12,52]. In contrast, the C3 orotype, characterized by the absence of *Neisseria* and *Haemophilus*, showed broad depletion of these pathways, suggestive of a metabolic shift toward fermentative processes typical of anaerobic oral biofilms [59]. Together, these patterns suggest a combination of functional redundancy across shared core pathways and ecological specialization associated with dominant taxa, with additional redundancy likely present within orotypes due to species-level heterogeneity.

Although the identified orotypes were largely independent of measured clinical variables in this healthy cohort, this does not preclude biological or clinical relevance. Distinct microbiome configurations, for example, may influence resilience to ecological perturbations and susceptibility to dysbiosis. Furthermore, the enrichment of nitrate reduction and nitrogen metabolism–related pathways within the *Neisseria/Haemophilus*-enriched C2 orotype raises the possibility that distinct tongue microbiome community states may differ in their contribution to oral nitric oxide metabolism and, consequently, cardiovascular physiology and blood pressure regulation [12,52]. In addition, since the tongue dorsum represents a major oral biofilm reservoir, variation in orotype composition may also reflect broader differences in oral ecological balance, oral hygiene status, or susceptibility to oral and systemic diseases [7–12,46,47]. Nevertheless, these implications remain speculative, and longitudinal, disease-oriented studies integrating functional and clinical data are needed to clarify their significance.

The oral phageome identified in this study was dominated by double-stranded DNA bacteriophages consistent with previous studies of the human oral virome [18,60]. Among phages with annotated lifestyles, temperate phages were more prevalent than virulent phages, which is consistent with the stable and biofilm-rich nature of oral microbial communities, where lysogenic interactions are common [18,61,62]. Predicted bacterial host profiles derived from phage sequences, however, revealed community structures that were not fully concordant with the overall bacteriome composition. Among classified hosts, some taxa, such as *Mediterraneibacter,* were prominent despite not being detected among the dominant members of the tongue bacteriome. Conversely, genera that were important in structuring the tongue bacteriome, particularly *Neisseria* and *Veillonella,* were not prominently represented among predicted phage hosts.

Several factors may contribute to this discordance. Host prediction methods rely on reference-supported associations such as CRISPR spacer matches or genomic similarity, which may not fully capture the diversity of phage–host interactions present in natural communities [63,64]. In addition, many bacteriophages exhibit host ranges spanning multiple related taxa, and viral reference databases remain incomplete for numerous oral bacterial lineages [64,65]. Consistent with this limitation, a substantial fraction of predicted hosts remained unclassified across taxonomic levels, highlighting the still limited representation of oral bacteriophages and their hosts in current reference databases. Because *Mediterraneibacter* is primarily associated with the gut microbiome [66], its appearance among predicted hosts likely reflects reference-based host assignments derived from previously characterized phage–host relationships rather than the actual bacterial hosts present in the tongue environment [63]. It could also reflect broader host ranges. Accordingly, the phage-derived host clusters (HG1/HG2) should be interpreted as exploratory phage-associated ecological patterns rather than direct equivalents of the bacterial microbiome orotypes (C1–C3).

The current study has limitations. The primary limitation of this study is the moderate sample size and its derivation from a single-center cohort composed of an Arab population, which may limit the generalizability of the findings. Accordingly, the identified orotypes may not fully represent microbiome configurations in other populations. Indeed, studies have shown differences in enterotypes by geographical locations [67]. Therefore, validation of the findings in larger, geographically and ethnically diverse cohorts is warranted. Another limitation is that the present study is descriptive in nature with functional potential derived from shotgun metagenomic data rather than direct measurements of microbial activity, limiting mechanistic insights. Follow-up studies involving functional analyses, e.g. transcriptomics, metabolomics and/or proteomics, to ascertain the physiological relevance of the orotypes are needed. A further limitation is that due to the cross-sectional design of the current study, changes in orotypes over time could not be assessed. Therefore, prospective longitudinal cohort studies are warranted to investigate temporal dynamics and to identify factors influencing the stability of these orotypes. Finally, the study did not include an assessment of tongue coating phenotype, which may have contributed, at least in part, to the observed compositional structures.

Future studies are needed to further elucidate the ecological and clinical implications of tongue microbiome configurations. In particular, integration of spatially resolved approaches could help clarify how these orotypes relate to the structural organization of tongue biofilms. In addition, investigating their potential role in modulating responses to antimicrobial or biofilm-targeting therapies may provide insight into inter-individual variability in treatment outcomes.

## Conclusion

This study provides a comprehensive metagenomic characterization of the healthy tongue microbiome, revealing distinct microbial community configurations (‘orotypes’) that share conserved core functions but differ in taxonomic composition and metabolic potential. In parallel, the largely unclassified phageome and its associated host–phage interaction patterns underscore the complexity of viral–bacterial dynamics within the tongue ecosystem. Future longitudinal and disease-oriented studies, incorporating larger cohorts and improved phage and host annotation, are needed to determine whether specific tongue microbiome configurations are predictive of disease risk or represent early ecological transitions toward dysbiosis.

## Supplementary Material

Supplementary Material Supplementary materials.pdf

## Data Availability

The data that support the findings of this study are available upon reasonable request from the corresponding author, [AAA].
