# Supplementary material for: Metagenomic analysis of tongue samples from healthy subjects identifies distinct microbiome orotypes
Source: J Oral Microbiol. 2026 Jul 11;18(1):2687934. doi: 10.1080/20002297.2026.2687934 (PMC13360510; doi:10.1080/20002297.2026.2687934)
Supplement: Supplementary Material — s.pdf [file ZJOM_A_2687934_SM7501.pdf]

# Supplementary Materials

**Supplementary Table 1. Association of clinical factors with species-level DMM clusters (orotypes)**

| Variable | C1 (n=41) | C2 (n=28) | C3 (n=23) | p-value |
|---|---|---|---|---|
| **Gender** | | | | |
| Female | 17 (41.5%) | 15 (53.6%) | 9 (39.1%) | 0.508 |
| Male | 24 (58.5%) | 13 (46.4%) | 14 (60.9%) | |
| **Mouthwash** | | | | |
| No | 39 (95.1%) | 25 (89.3%) | 21 (91.3%) | 0.610 |
| Yes | 2 (4.9%) | 3 (10.7%) | 2 (8.7%) | |
| **Nitrate intake** | | | | |
| High | 8 (19.5%) | 9 (32.1%) | 4 (17.4%) | 0.364 |
| Low | 33 (80.5%) | 19 (67.9%) | 19 (82.6%) | |
| **Periodontitis** | | | | |
| No | 27 (65.9%) | 18 (64.3%) | 15 (65.2%) | 0.991 |
| Yes | 14 (34.1%) | 10 (35.7%) | 8 (34.8%) | |
| **Smoking** | | | | |
| No | 29 (70.7%) | 23 (85.2%) | 16 (69.6%) | 0.327 |
| Yes | 12 (29.3%) | 4 (14.8%) | 7 (30.4%) | |
| **Age** Median (IQR) | 41 (35–46) | 41.5 (35–46.2) | 42 (36–46.5) | 0.807 |
| **NIS** Median (IQR) | 7 (4–10) | 8.5 (5–12) | 7 (6–9.5) | 0.531 |
| **Plaque index** Median (IQR) | 0.1 (0.1-0.2) | 0.1 (0.0-0.2) | 0.1 (0.1-0.2) | 0.488 |

• Categorical variables shown as counts (% within cluster).

• Continuous variables shown as median (IQR).

• p-values: Chi-square, Fisher's Exact, or Kruskal–Wallis as appropriate.

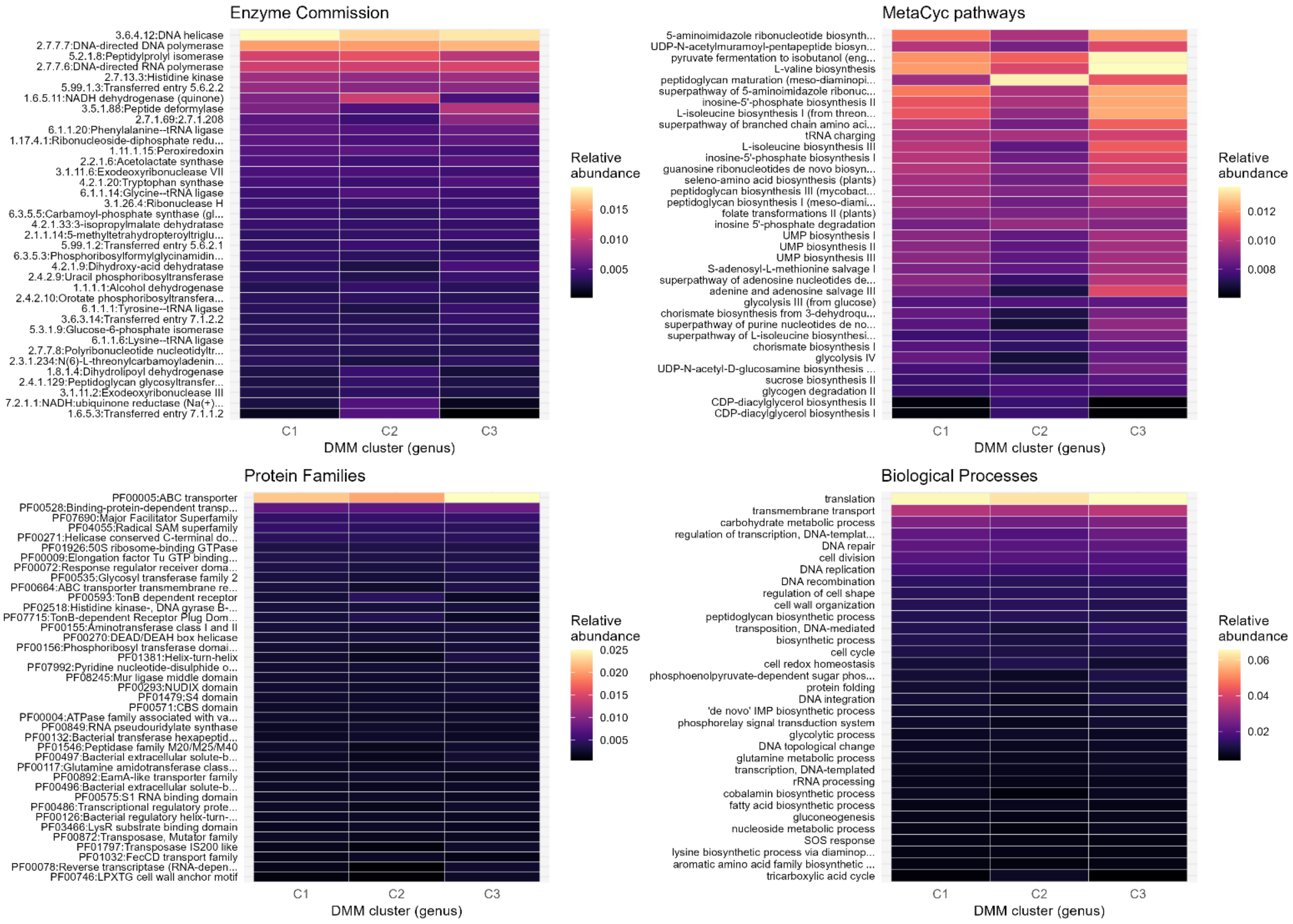


**Supplementary Figure 1. Dominant functional features across genus level microbiome orotypes.** Functional profiling of metagenomic data was performed using HUMAnN 3.0. Tile heatmaps show the mean relative abundance of the top 30 functional features within each genus-level DMM cluster (orotypes C1-C3) for **(A)** Enzyme Commission, **(B)** metabolic pathways, **(C)** protein families, and **(D)** Gene Ontology biological processes. Features were selected based on highest mean abundance within clusters. Feature names were truncated to 40 characters for readability.
